# Mycotoxin: Its Impact on Gut Health and Microbiota

**DOI:** 10.3389/fcimb.2018.00060

**Published:** 2018-02-26

**Authors:** Winnie-Pui-Pui Liew, Sabran Mohd-Redzwan

**Affiliations:** Department of Nutrition and Dietetics, Faculty of Medicine and Health Sciences, Universiti Putra Malaysia, Serdang, Malaysia

**Keywords:** mycotoxicosis, intestine, hepatocellular carcinoma, trichothecene, zearalenone, fumonisin, ochratoxin, aflatoxin

## Abstract

The secondary metabolites produced by fungi known as mycotoxins, are capable of causing mycotoxicosis (diseases and death) in human and animals. Contamination of feedstuffs as well as food commodities by fungi occurs frequently in a natural manner and is accompanied by the presence of mycotoxins. The occurrence of mycotoxins' contamination is further stimulated by the on-going global warming as reflected in some findings. This review comprehensively discussed the role of mycotoxins (trichothecenes, zearalenone, fumonisins, ochratoxins, and aflatoxins) toward gut health and gut microbiota. Certainly, mycotoxins cause perturbation in the gut, particularly in the intestinal epithelial. Recent insights have generated an entirely new perspective where there is a bi-directional relationship exists between mycotoxins and gut microbiota, thus suggesting that our gut microbiota might be involved in the development of mycotoxicosis. The bacteria–xenobiotic interplay for the host is highlighted in this review article. It is now well established that a healthy gut microbiota is largely responsible for the overall health of the host. Findings revealed that the gut microbiota is capable of eliminating mycotoxin from the host naturally, provided that the host is healthy with a balance gut microbiota. Moreover, mycotoxins have been demonstrated for modulation of gut microbiota composition, and such alteration in gut microbiota can be observed up to species level in some of the studies. Most, if not all, of the reported effects of mycotoxins, are negative in terms of intestinal health, where beneficial bacteria are eliminated accompanied by an increase of the gut pathogen. The interactions between gut microbiota and mycotoxins have a significant role in the development of mycotoxicosis, particularly hepatocellular carcinoma. Such knowledge potentially drives the development of novel and innovative strategies for the prevention and therapy of mycotoxin contamination and mycotoxicosis.

## Background

The momentum of scientific paper publication toward mycotoxin is an increasing trend where 16,821 papers were recorded in Scopus since the first mycotoxin, aflatoxin (AF) was identified in the year 1965. Data clearly showed the significance of mycotoxin research which will be further discussed later in this review paper. Nevertheless, the global health issue arose from mycotoxin is still frequently ignored in many low-income countries, where mycotoxins affect staple foods (Wild and Gong, 2010). The exposure is long-term and often at high doses, regretfully these particular regions are the least regulated in terms of agricultural practices and human exposure. The attention only has been paid in the richer nations of the world, to meet stringent import regulations on mycotoxin contamination (Battilani et al., 2016). To date, the world still desires for a more accurate evidence-based on mycotoxins and human health, as well as a better biomarker of exposure and data from studies of disease distribution. Current data are valid to justify and respond to reduce exposure in vulnerable populations (Freire and da Rocha, 2017). The implementation of more practical and affordable mycotoxin removal techniques at the household level to effectively reduce exposure are becoming increasingly important. When mycotoxins are introduced into the organism from food, they first come to interact with the gastrointestinal (GI) tract (Assunção et al., 2016). The GI tract is where the gut microbiota resides: it is known for its role in modulating the immune system and digestive processes. Gut microbiota work in concert with the GI tract protects the host from the toxicity of mycotoxins. Accordingly, integration of microbial-based approaches through maintaining a healthy gut microbiota is highly demanded.

## Mycotoxins

Mycotoxins, the low molecular mass (MW ~700 Da) secondary metabolites mainly produced by *Aspergillus, Penicillium*, and *Fusarium* are highly noxious substances on animals and humans. However, not all mycotoxin are classified as such, for example, Penicillin, is widely used an antibiotic (Speight, 2012). The structural form of mycotoxins varies from simple four C compounds, e.g., moniliformin, to complex substances such as the phomopsins (Zain, 2011). Fungal proliferation and production of mycotoxins rise naturally due to environmental factors, especially during tropical conditions (Mohd-Redzwan et al., 2013). Besides, the downstream processing such as poor harvesting practices, improper storage and less than optimal conditions during transportation, processing, and marketing can also contribute to the growth of fungi and increase the risk of the major food spoilage agent caused by mycotoxin production (Khazaeli et al., 2014). Due to their ubiquitous nature of fungi, mycotoxins have been increasingly attracting the concern of health organizations where their occurrence in foods cannot be ignored and already poses risk to consumers (Jahanian, 2016).

## Importance of research on mycotoxin

### Occurrence of mycotoxicosis

Notably, it has been estimated that 25% of the world's crops such as nuts, cereals, and rice are contaminated by mold and fungal growth, as reviewed by the United Nations Food and Agriculture Organization and the World Health Organization (Pandya and Arade, 2016). The toxic effect of mycotoxins on animal and human health is referred to as mycotoxicosis. Exposure to mycotoxins is mostly by ingestion but also occurs by the dermal and inhalation routes. The extent of adverse effects of mycotoxins on human or animals health mainly depends on the extent of exposure (dosage and period), type of mycotoxins, physiological and nutritional status as well as possible synergistic effects of other chemicals to which the animals or humans are exposed (Gajecka et al., 2013). In 1960, interest on mycotoxins was initiated by the occurrence of Turkey X disease caused by AF, which killed more than 100,000 turkeys. Subsequently, it was found that AFs are carcinogenic and cause hepatocellular carcinoma (HCC) in animals and humans, and this has stimulated research on mycotoxins (Peraica et al., 1999). Since then, around 400 mycotoxins are known, but AFs, ochratoxins, zearalenone (ZEA), fumonisins (FBs) and trichothecenes are mostly focused on public health issues (Ates et al., 2013). Mycotoxin exposure is not only limited to pure mycotoxins but also masked mycotoxin which formed when plants protect themselves by conjugating mycotoxins to biopolymers. In addition, some people are more susceptible to getting mycotoxicosis than others, and this is due to the pharmacogenetic variability where specific gene mutations such as cytochrome p450 (CYP 450) genes could either increase or decrease the metabolic activity (cytotoxicity) of the challenging mycotoxins (Sun et al., 2016). For instance, in both *in vivo* (Muhammad et al., 2017) and *in vitro* (Lewis et al., 2000) studies, CYPs' 1A2 and 3A4 appear as the most important enzymes that increased metabolism of AFB1 to its active form, AFB1-8,9-epoxide and subsequently to AFB1-DNA adduct formation, in which the biomarker has been linked to the development of liver cancer (Ceccaroli et al., 2015).

Chronic mycotoxicosis causes a greater impact on human health. Mycotoxin can induce diverse and powerful toxic effects in test systems: some are carcinogenic, mutagenic, teratogenic, estrogenic, hemorrhagic, immunotoxic, nephrotoxic, hepatotoxic, dermatoxic and neurotoxic (Milićević et al., 2010). Frequently, mycotoxicosis remains unrecognized by medical professionals. Mycotoxicosis can be weighed when a disease appears in several persons, with no obvious connection to a known etiological agent, such as microorganisms (Viegas et al., 2015).

### Future prospect: impact of growing population and ongoing climate change on mycotoxin

By the year 2030, the world's population is estimated to reach 8.2 billion people, and with 842 million people estimated as having been undernourished in the period of the year 2011–2013, food supply will definitely present a growing challenge in the next decades (FAO, 2014). This scenario will, in turn, have a tremendous negative impact on food supply (FAO, 2014). It is worth to note that the presence of hazardous substances (e.g., mycotoxins) also limits or reduces the marketability of food products in international markets (Anater et al., 2016).

There is now widespread consensus that the earth is warming at an unprecedented rate (Medina et al., 2015). The geographic distribution and production of the crop, as well as the phyllosphere microflora of crops, are expected to be strongly affected by climate change. For instance, mycotoxigenic *Aspergillus flavus* are able to grow under high temperatures and drought conditions. The resilient growth of *A. flavus* under extreme heat and dry condition is an expected and emerging dilemma mainly in the Mediterranean and other temperate regions (Logrieco et al., 2003). For example, the impacts of climate change have been observed in Serbia, where no contamination occurred previously, but prolonged hot and dry weather in the year 2012 resulted in 69% of maizes contaminated with AFs (Medina et al., 2015). A similar case also found in Hungary, where the increase in AFs contamination may be due to climate change conditions (Dobolyi et al., 2013).

The world's largest agri-food exporters include countries such as Brazil and Argentina and parts of Asia including China and India are identified as hot spots for impacts of climate change (Ray et al., 2012). Thus, from a food security perspective, a more accurate prediction of impacts of climate change on mycotoxins need to be addressed to prevent compromised food sustainability which possibly resulting in negative social consequences.

## Gastrointestinal tract

The GI tract is an organ within humans and other animals which responsible for food ingestion, digestion, energy and nutrients absorption, immune response, as well as elimination of waste products (feces) (Celi et al., 2017). The architecture of the GI tract is intended to facilitate these functions. The basic feature of GI tract is a muscular tube lined by a mucous membrane and comprised four layers forming a continuous passage. All segments of the GI tract are divided into four layers: mucosa, submucosa, muscularis propria, and serosa (Jaladanki and Wang, 2016). The mucosa is made up of three layers (epithelium, lamina propria, and muscular mucosae). The entire mucosa rests on the submucosa, beneath which is the muscularis propria. The outermost layer is named as the serosa. The complex infolding at mucosa layer forms an immense surface area for the most efficient nutrient absorption. The submucosa contains arteries, veins, inflammatory cells, lymphatics, and autonomic nerves. The muscularis mucosa is a thin layer of smooth muscle that forms the basis of peristalsis. While, the serosa is made of connective tissue that contains blood vessels, nerves, and fat (Jaladanki and Wang, 2016).

The epithelium layer at the innermost of mucosa is of vital importance for intestinal barrier function. The intestinal epithelium is one layer of thin cells lining the gut lumen. The epithelial contains enterocytes, enteroendocrine, and goblet cells at villi, whereas the Paneth cells, located under the crypts (Fink and Koo, 2016). It acts as a barrier to block the entry of harmful agents such as pathogens, toxins, and foreign antigens. Besides, it is also an important site for nutrient absorption including electrolytes, dietary nutrients, and water via its selective permeable membrane (Constantinescu and Chou, 2016). Each intestinal epithelial cell is connected by desmosomes, tight junctions (TJs), and adherens junctions (AJs). The AJs and desmosomes are responsible for the mechanical linkage of adjacent cells. Whereas, the TJs control the intercellular space and regulate selective paracellular ionic solute transport (Capaldo et al., 2014). Above the epithelium lies a complex microflora which is recognized as gut microbiota and the role of gut microbiota will be discussed later in this review article. The selective permeable barrier of mucosal epithelium establishes the interplay between the intestinal immune system and the luminal contents.

### Mycotoxins and gut health

Upon ingestion of contaminated food or feed, the GI tract is particularly affected by mycotoxin. Generally, intestinal barrier in the GI tract functions as a filter against harmful mycotoxins. However, some mycotoxins have been found to exert their detrimental effects in the GI tract. For example, mycotoxins can alter the normal intestinal functions such as barrier function and nutrient absorption. Some mycotoxins also affect the histomorphology of intestine. The impacts of mycotoxins include trichothecenes, zearalenone, fumonisins, ochratoxins, and AFs on general and gut health will be comprehensively reviewed.

#### Trichothecenes

*Fusarium graminearum* is the main fungi species that produces tricothecenes. All tricothecenes contain an epoxide at the C12, C13 positions, which is responsible for their toxicological activity (Nathanail et al., 2015). T-2 toxin (Type A) and DON (Type B) are the major mycotoxins that cause toxicity to humans and animals via oral ingestion (Nathanail et al., 2015).

During World War II, a biological weapon caused an acute syndrome consists of cough, sore throat, dyspnea, bloody nasal discharge, and fever was reported by Soviet scientists (Pitt and Miller, 2016). Twenty years later, T-2 mycotoxin was discovered when civilians consumed wheat that was unintentionally contaminated with Fusarium fungi (Pitt and Miller, 2016). A human toxicosis due to ingestion of moldy rice contaminated with T-2 toxin has been reported in China. According to Wang Z. et al. (1993), 65% of patients developed food poisoning symptoms such as chills, nausea, abdominal distension, dizziness, vomiting, thoracic stuffiness, abdominal pain, and diarrhea. Similar to T-2 toxicity, victims of DON outbreak suffered from vomiting syndromes (Etzel, 2014). Several outbreaks of acute DON toxicity in human have been reported in India, China, and the USA (Etzel, 2014).

Trichothecenes toxic effects in animals (dairy cattle, swines, broilers, and rats) include decreased plasma glucose, reduced blood cell and leukocyte count, weight loss, alimentary toxic aleukia, as well as pathological changes in the liver and stomach (Adhikari et al., 2017). The mechanism involved in T-2 and DON toxicity is generally via oxidative stress-mediated deoxyribonucleic acid (DNA) damage and apoptosis (Wu et al., 2014). Furthermore, T-2 and DON are well-known inhibitors of protein synthesis resulting from the binding of peptidyl-transferase, which is located in the 60s ribosomal subunit (Yang et al., 2017).

In the GI tract, a decreased absorption of glucose was observed following T-2 and DON intoxication resulted from suppressed SGLT1 (glucose transporter) mRNA expression. Apart from the glucose absorption, SGLT1 also responsible for water reabsorption, thus reduction of SGLT1 transporter induces diarrhea as well (Grenier and Applegate, 2013).

The weight loss effect of trichothecenes involved neuroendocrine factors and cytokines. DON and T-2 elevated concentrations of the indoleamines, serotonin and 5-hydroxy-3-indoleacetic acid (HIAA) in all brain regions (Wang J. et al., 1993). These neuroendocrine factors can affect the secretion of both anorexigenic and/or orexigenic hormones (Maresca, 2013). Through increasing gene expression of anorexia-inducing proinflammatory cytokines such as interleukin-1β (IL-1β), interleukin-6 (IL-6) and tumor necrosis factor-α (TNF-α), trichothecenes exacerbate the condition of anorexia (Wu et al., 2015). In addition, DON and T-2 also induced the release of the satiety hormones, peptide YY (PYY) and cholecystokinin (CCK), which are critical mediators of anorexia (Wu et al., 2015).

Using animal models, trichothecenes was found to induce necrotic lesions in the GI tract (Kolf-Clauw et al., 2013). A shortening of villi height was also observed in trichothecenes-treated animals (swine, poultry, and rat model). The changes on villi were due to activation of the apoptotic pathway by trichothecenes, which in turn leads to nutrition malabsorption (Alizadeh et al., 2015). Furthermore, results obtained from *in vivo* and *in vitro* studies showed that trichothecenes increased intestinal permeability. Using porcine epithelial cell, trichothecenes increased the intestinal permeability by lowering tight junction proteins expression (Osselaere et al., 2013). In addition, previous studies revealed a significant (*P* < 0.05) decreased in the number of goblet cells that secrete mucin in trichothecenes-treated animals. Mucin is primarily involved in the gut barrier function (Pinton and Oswald, 2014). The disruption in the integrity of intestinal epithelium allows the entry of the pathogen into the gut lumen (Lessard et al., 2015). Besides, trichothecenes have been linked with a decreased level of IL-8 in the intestine, which is responsible for pathogen removal (Kadota et al., 2013). Overall, trichothecenes exert negative impacts on GI tracts specifically on the gut absorption, integrity, and immunity.

#### Zearalenone

Zearalenone (ZEA) is a mycotoxin that primarily produced by *Fusarium graminearum* and *Fusarium culmorum* in foods and feeds. The high rate of co-occurrence of ZEA with FBs and DON indicates that these mycotoxins might be involved in a wide range of synergistic and additive interactions. ZEA has been linked to scabby grain toxicosis occurred in Japan, China, Australia, and the USA, with symptoms including nausea, vomiting, and diarrhea (Liao et al., 2009).

It is well recognized that ZEA is a non-steroidal estrogenic mycotoxin that is implicated in the reproductive disorders of farm animals (swines, cattle, and sheep) and hyperoestrogenic syndromes in humans (Kotowicz et al., 2014). Toxicological studies of ZEA revealed its effects on the reproductive system, including enlarged uterus, altered reproductive tract, decreased fertility, as well as abnormal level of progesterone and estradiol. Besides, the ingestion of ZEA during pregnancy reduced fetal weight and survival rate of embryo (Zhang et al., 2014). This phenomenon can be explained through the structure of ZEA. ZEA has a structure which allows it to bind to the mammalian estrogen receptor, although with lower affinity compared to the natural-occurring estrogens (Hueza et al., 2014). Besides, ZEA has also been shown to be hepatotoxic, haematotoxic, immunotoxic and genotoxic (Zhou et al., 2017).

Although the reproductive organ is the main target for ZEA to induce toxicity, the adverse effects of ZEA on GI tracts have been reported. The effects of ZEA ingestion on the GI tract are not as detrimental compared to the other mycotoxins. Studies using intestinal epithelial cells showed that ZEA induced cell death without altering the cell integrity as indicated by transepithelial electrical resistance (Marin et al., 2015). In contrast, it was discovered that the metabolites of ZEA (α- and β-zearalenol) significantly (*P* < 0.05) decreased the cell integrity. The study showed that ZEA and its metabolites acted differently in the gut (Marin et al., 2015). Abassi et al. (2016) demonstrated that ZEA enhanced cell proliferation, increased colony formation and fastened cell migration of colon carcinoma cell line HCT116. Another study also showed that ZEA down-regulated the expression of tumor-suppressor genes (PCDH11X, DKK1, and TC5313860) in intestinal cells (Taranu et al., 2015). In fact, the modulation of gene expression was responsible for the carcinogenic effects of ZEA. Nevertheless, swines ingested ZEA did not showed changes in the height of villi, the thickness of the mucosa, and number of goblet cells (Gajecka et al., 2016a,b; Lewczuk et al., 2016). In brief, ZEA plays a negative role in gut health although no apparent histological changes have been observed.

#### Fumonisins

*Fusarium verticillioides* is the major producer of fumonisins, where fumonisin B1 (FB1) is the most abundant in nature (Lerda, 2017). In contrast to most mycotoxins, which are hydrophobic in nature, fumonisins are hydrophilic compounds, which hinders its discovery until 1988 (Gelderblom et al., 1988). Human epidemiological studies in South Africa, Italy, and China revealed that the esophageal cancer is related to the intake of corn grains containing fumonisins (Chilaka et al., 2017). Another epidemic of neural tube defects (birth defects of the brain, spine, or spinal cord) occurred along the Texas-Mexico border, China and South Africa were also found to be associated with fumonisins-contaminated corn consumption (Ortiz et al., 2015). In animals, fumonisins have been found to cause pulmonary edema and hydrothorax in swines; leukoencephalomalacia in equine; and HCC in rats (da Rocha et al., 2014).

FB1 shares the same structure as cellular sphingolipids (Masching et al., 2016). Sphingolipids are responsible for neurological and immunological diseases, as well as cancer. Normal degradation of sphingolipids to ceramide requires sphingomyelinase and ceramidase (Boini et al., 2017). However, FB1 disturbs sphingolipids metabolism via ceramide synthase inhibition which leads to sphingosine accumulation in cells (Masching et al., 2016). FB1 elevates sphingosine levels in urine, serum, kidney, liver, and small intestine. The abnormal turnover of sphingosine induced cytotoxicity, oxidative stress, apoptosis in cells (Hahn et al., 2015).

Using intestinal cell lines (IPEC-1, Caco-2, and HT29), Minervini et al. (2014) found FB1 decreased the cell viability and proliferation in a concentration-dependent manner. A possible mechanism has been suggested through the accumulation of sphinganine by FB1. In the intestinal epithelial cells, sphinganine accumulation blocked G0/G1 phase in cell and resulted in growth inhibition and apoptosis (Angius et al., 2015). The accumulation of sphinganine also altered glycoprotein distribution in the jejunum and caused an increase in transepithelial passage of FB1 (Yamazoe et al., 2017). In addition, FB1 altered the integrity of intestinal barrier by suppressing tight junction (TJ) protein expression level (Romero et al., 2016). The increase in intestinal permeability, in turn, promotes translocation of bacteria (Kelly et al., 2015).

Besides, high levels of FB1 also induced an overgrowth in intestinal goblet cell of broiler and swine (Alassane-Kpembi and Oswald, 2015). Goblet-cell hyperplasia is associated with increased mucin secretion. However, continuous hypersecretion of mucins might deplete the number of goblet cells, resulting in devastation of mucus barrier (Johansson and Hansson, 2016). Previous studies conducted using intestinal cell lines (IPEC-1, Caco-2, and HT29) showed that FB1 was able to regulate immune responses. Upon LPS exposure to the FB1-treated cell line, a reduction in IL-8 synthesis was detected (Minervini et al., 2014). Such reduction could be responsible for a low number of polymorphonuclear leukocytes (PMNs) recruited to infection sites, thus leading to the ineffective elimination of pathogen from the gut (Brazil et al., 2013). Generally, in the gut, FB1 increased intestinal cell apoptosis, reduced intestinal barrier and caused immune dysfunction.

#### Ochratoxin

Ochratoxin is mainly produced by *Aspergillus species* and *Penicillium species*. Ochratoxin A (OTA) is the most prevalent and relevant fungal toxin of this group (Liuzzi et al., 2017). The main target site of OTA is kidney. Previous findings from animals showed OTA is a potent renal carcinogen (Russo et al., 2016). The International Agency for Research on Cancer (IARC) categorized OTA as possibly carcinogenic to humans under Group 2B carcinogen. Apart from that, OTA is an immunosuppressive, teratogenic, and nephrotoxic compound (Ladeira et al., 2017).

In human studies, OTA is associated with kidney diseases, such as Balkan endemic nephropathy (BEN). BEN is a chronic tubulointerstitial disease which slowly progressed into terminal renal failure. Indeed, a 15 years study confirmed that BEN is correlated with upper urothelial tract cancer (Rouprêt et al., 2015). Furthermore, OTA has been associated with the occurrence of upper urothelial tract cancer (Fahmy et al., 2014). However, a systemic review by Bui-Klimke and Wu (2015) revealed that there is no significant evidence for human health risks associated with OTA exposure based on the epidemiological data. The modes of toxic action of OTA are identified through the blockage of protein synthesis and energy production, the formation of DNA adduct formation, apoptosis, as well as the induction of oxidative stress (Kőszegi and Poór, 2016). Moreover, recent studies showed OTA triggered autism via epigenetic mechanism (Mezzelani et al., 2016).

Other than its adverse effects on the kidney, previous studies also revealed the gut changes induced by OTA. OTA altered nutrition absorption in the intestine. *In vitro* studies demonstrated that OTA decreased glucose absorption via SGLT1 transporter (Peraica et al., 2011). In addition, OTA-treated animals experienced faster and more harmful parasite infections (provoked by *Eimeria acervulina* and *E. adenoeides*) in chicks and turkey compared to control. The results showed that animals fed with OTA had higher lesion and oocyst indexes in the intestine and more damage at mucosa (Manafi et al., 2011). This can be explained by the increased intestinal permeability in the presence of OTA. In addition, results from immunoblotting and immunofluorescence showed that the expression of TJ proteins responsible for intestinal integrity was significantly (*P* < 0.05) suppressed by OTA (McLaughlin et al., 2004). Besides, OTA-induced oxidative stress also can alter intestinal permeability (Anderson et al., 2016). The oxidative stress induced by OTA has been found to be associated with the apoptosis in the intestinal IPEC-J2 cells (Wang et al., 2017). A study by Solcan et al. (2015) on OTA-fed broilers revealed there is a decrease in villi height and increase in apoptosis of intestinal epithelial cells. Similar results were obtained from another study using broiler model (Qu et al., 2017). Inflammation pathway in the intestine was also affected by OTA. The expression of inflammation-related cytokines (IL-8, IL-6, IL-17A, IL-12, and IL-18) was significantly (*P* < 0.05) decreased in the intestine of the piglets exposed to the toxin (Marin et al., 2017). The alteration of immune system renders the gut vulnerability to infection. OTA exerted its effect on gut via the reduction of nutrient absorption, disruption of intestinal permeability, cell apoptosis, and modulation of immune system.

#### Aflatoxin

AF is a mycotoxin produced by *Aspergillus flavus* and *Aspergillus parasiticus*. The most common mycotoxin found in human food and animal feed is AFB1. In fact, AFB1 is the most potent hepatocarcinogen recognized in mammals and listed as Group I carcinogen by IARC (Muhammad et al., 2017). Liver is the main target site of AFB1. Cumulative evidences from human and animals revealed a strong linkage occurs between AFB1 and HCC. While, acute aflatoxicosis induced abdominal pain, vomiting, edema, and death (Mohd-Redzwan et al., 2013).

Aflatoxicosis outbreak has been recorded four times in Kenya from 2004 to 2014, with near to 600 individuals were affected and 211 deaths were reported from the tragic outbreak (Awuor et al., 2017). As discussed earlier, p450 enzymes in the liver metabolize AFB1 into AFB1-8,9-exo-epoxide. The highly reactive exo-epoxides form derivatives with DNA, RNA and proteins which subsequently react with the p53 tumor suppressor gene. The reaction generates AFB1-N7-Gua which is then converted to its stabilized form, AFB1-formamidopyrimidine (AFB1-FABY) adduct. AFB1-FABY causes transversion of guanine (G) to thymine (T), which leads to mutation and malignant transformation (Kew, 2013). In addition to the hepatotoxicity of AFB1 mentioned above, other adverse effects include growth retardation, immunosuppression, and genotoxicity have been reported (Kumar et al., 2017).

Like most of the mycotoxins, AFB1 compromised the health of GI tracts. Colon cell line (Caco-2) was used in *in vitro* experiment to determine the AFB1 toxicity in the intestine. AFB1 significantly (*P* < 0.05) inhibited cell growth, increased lactate dehydrogenase activity and caused genetic damage. It is found that the mechanism of AFB1 cytotoxicity are associated with reactive oxygen species (ROS) generation, which leads to the damage of cell membrane and DNA (Zhang et al., 2015). Besides, transepithelial electrical resistance assay showed a reduction in intestinal Caco-2 cells' integrity after AFB1 treatment (Romero et al., 2016).

Similar result has been observed in *in vivo* study where AFB1 affects intestinal barrier function in broiler model as indicated by an increased ratio of lactulose to rhamnose ratio in the plasma (Chen et al., 2016). Several studies on broiler exposed to AFB1 showed that the density (weight/length) of intestine was reduced (Hossein and Gurbuz, 2015). An increase of apoptotic events was found in the jejunum, accompanied with elevated apoptotic markers (Bax and caspase-3) mRNA expression level. Moreover, the increased apoptosis was corresponded to a lower jejunal villi height as found in the other studies (Peng et al., 2014; Zheng et al., 2017). Another study by Akinrinmade et al. (2016) demonstrated intestinal injuries induced by AFB1 in rats. In the AFB1-fed rat, leucocyte and lymphocyte infiltration were observed at lamina propria of the intestinal mucosa. In the duodenum and ileum, AFB1 exposure caused intestinal lesions such as the development of sub-epithelial space and villi degeneration. The adverse effects on the gut from AFB1 exposure include the disruption of intestinal barrier, cell proliferation, cell apoptosis, and immune system. Although AFB1 is the most life-threatening mycotoxin, yet its toxicity on the gut is comparable to the other mycotoxins.

## Gut microbiota

The gut microbiota represents an ensemble of microorganisms including bacteria, viruses, and fungi that harbor within the GI tracts of living organisms. In the past, gut microbiota studies have been focused on the association of single pathogenic organisms with human health. Non-pathogenic microbes were previously thought to be benign as compared to the pathogens (Holmes et al., 2011). Nevertheless, the gut microbiota has recently become a blooming research area (Hoffmann et al., 2013).

The rapid rise of remarkable and cost-effective next-generation DNA sequencing methods provide an effective approach to study the composition of the host microbiota. Metagenomic sequencing and amplicon sequencing using specific genes markers are established to replace culture-independent methods for host microbiota analysis (Kuczynski et al., 2012). The amplified sequences resulting read abundance, which reflects the microbial diversity. The advances in molecular biology have provided innovative ways to entangle the complex microbial communities. Using the methods, it has been revealed that majority of microorganisms resides in the gut cannot be cultured outside the host. In the human, for instance, approximately 80% of the total bacterial species in the gut failed to be cultured under laboratory conditions (Guarner and Malagelada, 2003). Besides, these methods also revealed differences in gut bacterial community between anatomical sites, between individuals, and between healthy and diseased states. The findings have completely transformed the view of mammals biology (Weinstock, 2012). As such, growing interest has led to an increasing research into the communities of non-pathogenic microbes that inhabit the human body, and the need to describe the genomes of these organisms to understand the human microbiota has been recognized.

The composition of the gut microbiota varies significantly at the relative ratios of dominant phyla, genera, and species. In particular, stable and healthy gut microbiota is generally indicated by the rich diversity of gut bacteria (Mosca et al., 2016). The rapid development of gut microbiota study has revealed its significant role in maintaining human health. The involvement of gut microbiota in nutrition, metabolism, and immune function has been well established. The gut microbiota allows the host to metabolize a vast range of dietary substrates. For example, the metabolism of carbohydrate is a major catalytic function of the microbiota. The gut microbiota (specifically *Bacteroides, Bifidobacterium, Enterobacterium, Fecalibacterium*, and *Roseburia*) assist in the fermentation of complex polysaccharides that escaped proximal digestion (Jandhyala et al., 2015). The fermentation process produces monosaccharides and short chain fatty acids (SCFAs) which include acetate, butyrate, and propionate that are rich energy sources for the host (Jandhyala et al., 2015). Similarly, metabolism pathways of proteins, bile, and phytochemicals, as well as vitamins synthesis by the microbiota, has been also elucidated (Rowland et al., 2017).

Apart from that, the gut microbiota protects the host against infections via several mechanisms. The microbes reside in the gut modulate the population of pathogenic microorganisms via competitive exclusion for attachment sites and nutrient (Donaldson et al., 2016). The significance of gut microbiota in the development of immunity can be readily appreciated from the study of germ-free (GF) mice. By comparison to normal mice, GF mice which have a lack of microbiota have been shown to exhibit irregularities in cytokines profile, contain poorly formed local and systemic lymphoid structures as well as the abnormal level of immune cells (Sekirov et al., 2010). Furthermore, accumulating recent data demonstrated that the functions of gut microbiota further extend beyond the gut. Mechanisms studies suggesting that microbial metabolites have taken the role by sending signals to peripheral organs, including the liver, adipose tissue, pancreas, cardiovascular system, lung, and even to the brain (Lv et al., 2017).

It is widely recognized that the composition of gut microbiota in newborns is obtained from mothers during delivery (Dominguez-Bello et al., 2010). While the alteration in the composition of the gut microbiota is mediated by numerous factors including dietary changes (Cani and Everard, 2016), development of disorders and diseases (Hand et al., 2016), genetics as well as stressful experiences (Karl et al., 2017). Sufficient evidence has revealed the inter-relationship between dietary habit and the intestinal microbiota composition (Cani and Everard, 2016). For example, high fat diet renders the host to harbor a gut microbiota enriched in the phylum *Firmicutes* and depleted in *Bacteriodetes*. Besides, high fat diet has been also identified to promote proliferation of specific bacterial strains such as Enterobacteriaceae, which may increase intestinal lipopolysaccharide and subsequently increase gut permeability as well as triggering inflammation (Bibbò et al., 2016). On the other hand, a diet rich in fiber has been observed to modulate gut microbiota by altering fermentative metabolites and intestinal pH. Fermentation of fiber by the colonic microbiota produces SCFAs, wherein the metabolites play a significant role in regulating pH in the intestine. It has been demonstrated that a decrease in pH is able to significantly (*P* < 0.05) decrease the population of Bacteriodetes spp. and members of Enterobacteriaceae while promoting the growth of beneficial butyrate-producing microorganisms (Duncan et al., 2009).

On the other hand, studies revealed that the host genotype contributes remarkably to the resemblances in the gut microbial taxa. The genes linked with microbial taxa are particularly responsible for diet sensing, immunity, and metabolism (Goodrich et al., 2016b). Moreover, data showed a family belongs to the Firmicutes and Christensenellaceae has the highest heritability. The presence of Christensenellaceae in the gut is frequently associated with low serum triglyceride levels observed in lean and healthy human phenotype (van Opstal and Bordenstein, 2015). In genotype factor studies, monozygotic twins which developed from one zygote are often used as the subjects. As compared to dizygotic twins, it has been shown that there is a higher carriage of *Methanobrevibacter smithii* in monozygotic twins. *M. smithii* has also found to be related with leanness (Goodrich et al., 2016a). Besides, numerous studies have linked genetic loci with population of gut bacteria in mice and humans. For instance, *LCT* gene which encodes for lactase-phlorizin hydrolase. Single nucleotide polymorphisms (SNPs) in *LCT* are directly correlated with lactose intolerance and the abundance of lactose-metabolizing bacteria, specifically *Bifidobacterium* (Lerner et al., 2017).

Recently, the occurrence of diseases has often been linked consequentially to dysbiosis of gut microbiota. A wide range of gut microbiota-related diseases have been revealed include autism, asthma, colon cancer, Crohn's Disease, irritable bowel syndrome (IBS), food allergies, cardiovascular disease, obesity, diabetes, eczema and hepatic encephalopathy, mental disorders (Kamada et al., 2013; Sommer and Bäckhed, 2013; Korem et al., 2015). For instance, bacterial overgrowth in the small intestine is commonly observed in patients with IBS. An investigation using 16S rRNA-based microbiota profiling approaches on IBS subjects revealed quantitative and qualitative changes in both mucosal and fecal gut microbiota (Simrén et al., 2012; Collins, 2014). The findings suggested that the gut microbiota balance was compromised in the events of gut inflammation. The dysbiosis of gut microbiota initiates mucosal innate immune responses and increases intestinal permeability. Subsequently, translocation of pathogens occurs, and harmful metabolites can enter the intestinal epithelium. Such events in the gut further exacerbate the severity of diseases (Collins, 2014). Apart from that, evidence showed that the gut microbiota-derived products (SCFA, neurotransmitters, enzymes, and toxins) can be absorbed from the gut, and subsequently affect the metabolic phenotype of the host (Lee and Hase, 2014). In addition, metabolites from the host are transported into the gut via the enterohepatic circulation and serve as substrates for the microbiota. These processes give rise to an interspecies cross-talk between the host genome and the gut microbiota (Lee and Hase, 2014).

Regrettably, fewer studies have discussed on the importance of the minorities such as virus, commensal fungi, archaea, and protozoa. The gut virome comprised plant-derived viruses, giant viruses, and abundant (90%) bacteriophages. Pathogenicity of gut viruses includes gastroenteritis, pneumonitis, and diarrhea (Scarpellini et al., 2015). Whereas, fungal communities in the gut mainly consist of the Ascomycota, Basidiomycota, and Zygomycota. *Candida* species have been primarily associated in inflammatory bowel diseases (IBD), Crohn's disease (CD), ulcerative colitis (UC), obesity and gut inflammation (Sam et al., 2017). Undeniably, the gut microbiota influences almost all system resides in our body, which is of vital importance for survival, therefore, maintaining a balanced microbiota is essentially important.

### The bi-directional interaction between mycotoxin and the gut microbiota

Gut microbiota represents an important bridge between environmental substances and host metabolism. Findings found that gut microbiota, particularly in animals have profound interactions with ingested mycotoxins. Microbes reside in the gut aid host in the mycotoxin removal process through metabolizing or binding to the mycotoxins. Although some microbes possess the mycotoxin removal ability, it is noteworthy to mention that bacteria from the same genus, however, are unable to remove mycotoxin. Interestingly, few studies also demonstrated that mycotoxins can alter the gut microbiota. Such findings suggested that there is a bi-directional interaction occurs between mycotoxin and the gut microbiota. Evidence of disturbance on gut microbiota modulation induced by mycotoxin only had been studied on animal and the results have been summarized (Table 1). The changes in gut microbiota can be observed up to species level in some of the studies using advance molecular approaches. However, the compositions of gut microbiota are greatly influenced by various factors during the experiment. Confounding factors affecting microbial composition and function may include diet (Cani and Everard, 2016), the exposure of environmental chemical and antibiotics (Claus et al., 2017), genetic background (Goodrich et al., 2016b), as well as the mental health condition (stress) of the host (Karl et al., 2017). These factors can explain that the microbiota in same species may not be able to reduce the level of mycotoxins. Besides, the changes in gut microbiota due to the presence of mycotoxin may contribute by some uncontrolled variables. Contrasting data obtained from different studies in this review further highlight the significance of confounding factors toward the outcome of studies. Nonetheless, a well-controlled study designs is essential to ensure repeatable studies with consistent results.

**Table 1:** *In vivo* experiments: Gut microbiota alteration by mycotoxins.

**No**	**Subjects**	**Age**	**Comparison**	**No. of subjects**	**Treatment period**	**Methods**	**Taxonomic findings**	**Functional findings**	**References**
1	Male Swedish Landrace x Yorkshire pigs; SPF	Not specified, 20 kg	Post-valve T-caecum cannulas pig treated with 0.05 mg DON/kg BW /day: Before and after exposure to feces from pigs with DON de-epoxidation ability	5	Daily for 10 weeks	Terminal restriction fragment length polymorphism (T-RFLP)	No difference could be detected in the T-RFLP profiles in the intestinal microflora between samples from pigs before and after acquiring the ability to de-epoxidate trichothecenes.	The deoxynivalenol de-epoxidation ability was found in fecal and ileal, 1 week after the pigs were exposed to feces from pigs known to have the de-epoxidation ability.	Eriksen et al., 2002
2	Large White pigs; SPF	9-week-old	Healthy and DON (2.8 mg/kg) treated rat	24	Daily for 4 weeks	Capillary Electrophoresis Single-Stranded Conformation Polymorphism (CE-SSCP)	The species richness index was significantly increased by DON exposure.	A significant reduction of daily weight gain was observed in piglets exposed to the contaminated diet when compared to control.	Waché et al., 2008
3	Male Sprague-Dawley rats; GF	8 weeks old, 120–150 g	Germ-free rats inoculated with human fecal: Untreated and treated with DON (100mg/kg BW)	20	Daily for 4 weeks	Real-time PCR	DON significantly increased Bacteroides/Prevotella group and decreased concentration levels for *Escherichia coli*.	-	Saint-Cyr et al., 2013
4	Female Wistar rats; Pregnant	Not specified	Healthy pregnant rat fed with normal diet and DON-contaminated diet (10 mg/kg)	24	Daily for 4 weeks	16S sequencing	No difference could be detected in the intestinal microflora between control group and DO-treated group.	DON exacerbates the DNA damage induced by *E. coli* producing colibactin	Payros et al., 2017
5	Large-White piglets; SPF	4 weeks old, 41.6 kg	Healthy piglets fed with normal diet and diet containing 12 mg/kg of fumonisins (Mixture of FB1 and FB2)	48	Daily for 63 days	Capillary Electropho-resis Single-Stranded Conformation Polymorphism (CE-SSCP)	Fumonisins decreased the fecal microbiota SSCP-profiles similarity between the fumonisins treated and the untreated control group.	Fumonisins affected sphingolipid [sphinganine (Sa) and sphingosine (So)] metabolism.	Burel et al., 2013
6	Male Fischer 344 rats	6–7 weeks old	Healthy and OTA (70 and 210 μg/kg BW) treated rat	18	5 days/ week for 4 weeks	16S rRNA and shotgun sequencing	OTA increased Lactobacillaceae and decreased Bacteroidaceae in relative abundance. In genus level, Bacteroides, Dorea, Escherichia, Oribacterium, Ruminococcus, and Syntrophococcus were decreased and Lactobacillus increased. Facultative anaerobes were increased whereas anaerobes were reduced.	Changes in functional genes of gut microbiota including signal transduction, carbohydrate transport, transposase, amino acid transport system, and mismatch repair were observed.	Guo et al., 2014
7	Male Fischer 344 rats	5-weeks old, 120–130 g	Healthy and AFB1 treated rat; control, low (5 mg AFB1/kg BW), medium (25 mg/kg BW), high (75 mg/kg BW)	20	5 days/ week for 4 weeks	16S sequencing	Fecal: Clostridiales and Bacteroidales were increased in a dose-dependent manner of AFB1. In contrast, Lactobacillales from Firmicutes, Streptococcus sp. and Lactococcus sp. were decreased in a dose-dependent manner of AFB1 exposure.	-	Wang et al., 2015
8	Male Broiler chicks	1 day old	Healthy and AFB1 treated rat; control, 2 ppm, 1.5 ppm, 1 ppm daily	240	Daily for 21 days	Differential Agar (deMan Rogosa Sharpe for LAB; MacConkey for Gram-negative bacteria	AFB1 significantly reduced total LAB in chickens that received 1 ppm AFB1. In chickens fed with 2 and 1.5 ppm AFB1, the total number of Gram-negative bacteria and LAB were significantly increased.	AFB1 increased the heterophils-to-lymphocyte ratio. The villus length in both duodenum and ileum increased significantly by AFB1 as well as a reduction in duodenum crypt.	Schmeits et al., 2014
9	Female BALB/c mice	Not specified	Germfree and conventional microflora mice treated with AFB1 (10 mg/kg P.O) or dimethylsulfoxide (control)	12	1 h	CFU counts	Liver: Greater numbers of bacterial mutants were recovered from the conventional flora mice than from the germfree mice after AFB1 treatment.	Potentiated toxic effects.	Rowland, 1988
10	Gilts	25 ± 2 kg	Healthy and mycotoxin mixture treated gilts; control, 40 μg ZEA/kg BW + 12 μg DON/kg BW	75	Daily for 6 weeks	Differential Agar	ZEA and DON mixture significantly reduced amounts of *Clostridium perfringens, Escherichia coli*, and Enterobacteriaceae but increased the biodiversity.	Mixture of ZEA and DON increased amino acid metabolism in microbiota.	Piotrowska et al., 2014
11	Dairy calves	Less than 1 month and mature calf	Mycotoxin [aflatoxin (1–3 ppb) and fumonisin (50–350 ppb)] induced HE calf: Untreated and treated with Celmanax®/Dairyman's Choice™	From 3 production sites (Not specified)	Daily for 14 days	CFU counts	Fecal: Aflatoxin and fumonisin increased Shiga Toxin-producing *Escherichia coli* (STEC).	Aflatoxin exposure affects the STEC-secreted cytotoxin composition indicated by increasing intracellular Ca^2+^ concentrations corresponding to increased cytotoxicity.	Baines et al., 2013

#### Deoxynivalenol

A study has demonstrated the ability of gut microbiota to remove deoxynivalenol (DON) using an *in vitro* study (He et al., 1992). It was reported that the microorganisms in large intestines of broilers were able to transform DON into de-epoxy-DON. The gut microbes of broiler were further shown to transform DON to the less toxic metabolite, de-epoxy-DON via epoxide reductase. Similar findings have been observed in other studies using microbial content from the broiler intestine (Lun et al., 1988; Young et al., 2007). Some other studies have also shown that intestinal microorganisms of other animal species including rat (Worrell et al., 1989) and swine (Kollarczik et al., 1994) possess the same ability. However, no alteration was found when swines' intestines content was used in the study conducted by He et al. (1992).

Apart from these, mycotoxin degrading bacteria had been isolated from intestinal content for extensive studies. Microbiological selection strategies guided by PCR DGGE (denaturing gradient gel electrophoresis) had been employed to isolate DON-transforming bacteria and the isolates obtained belong to four different bacterial groups; Anaerofilum, Bacillus Clostridiales, and Collinsella (Yu H. et al., 2010). In addition, a microbial community, namely microbial culture C133 from catfish digesta was screened, and capable to completely transformed DON to de-epoxy-DON after 96 h incubation (Guan et al., 2009). Several intestinal bacterial strains have been identified as biological trichothecene detoxification agent via *in vitro* screening and microbial analyses in other recent reviews (Hathout and Aly, 2014). Interestingly, a study showed the ability to metabolize DON can be obtained via gut microbiota transfer in swine. However, the transfer of gut microbiota revealed no changes in the DNA-profiles of the gut bacterial composition (Eriksen et al., 2002).

Several studies have been done on the interaction of DON toward the gut microbiota. Consumption of feed contaminated with DON for a time duration of 4 weeks has been shown to exert minor effect on the total number of fecal aerobic mesophilic bacteria and anaerobic sulfite-reducing bacteria in swine. Although there was no effect of DON on microbial diversity, the richness index was significantly (*P* < 0.05) increased by DON exposure (Waché et al., 2008). In another study by Saint-Cyr et al. (2013), GF male rats were inoculated with fecal flora from healthy human, in order to investigate the human gut microbial changes induced by DON. By using real-time PCR quantification, a significant increase of Bacteroides/Prevotella group and decreased concentration levels of *Escherichia coli* were observed after feeding the rats with DON for 4 weeks (*P* < 0.05). In human, the shift in the proportion of Bacteroides is highly associated with diseases as individuals with Crohn's disease or celiac diseases often exhibit a higher abundance of Bacteroides than healthy individuals (Kamada, 2016). A recent study, however, revealed that DON has no significant changes in the diversity and relative abundance of gut microbiota based on 16S rRNA microbiota analysis (Payros et al., 2017).

#### T-2 toxin

Gratz et al. (2017) demonstrated that masked T-2 toxin was released as a parent mycotoxin by human gut microbiota, and thereby contribute to mycotoxin exposure. Trichothecene mycotoxins are generally known as ribotoxic stress inducer which effectively blocks eukaryotic 28S rRNA. Thus, in theoretical aspect, the T-2 toxin would not interfere with bacterial protein translation and growth as suggested by Schmeits et al. (2014). Nevertheless, this is in contrast to the observation seen in a study conducted by Tenk et al. (1982). It was shown that the administration of T-2 toxin for 1 week was sufficient to induce a substantial increase in the aerobic bacteria count in the intestine of swines and rats (Tenk et al., 1982). While the bacterial populations have been shown to be greatly affected by trichothecene, the mechanism which causes the perturbation of bacterial population remains to be elucidated.

#### Zearalenone

An *in vitro* experiment demonstrated that zearalenone compounds were converted into unknown metabolites by human gut microbiota (Gratz et al., 2017). The first study on the effect of ZEA on gut microbiota has been carried out by Piotrowska et al. (2014). The changes in gut microbiota were evaluated using Biolog EcoPlate method which only allows the quantification of culturable bacteria. After 6 weeks of ZEA ingestion, the data showed the concentration of *Clostridium perfringens, Enterobacteriaceae*, and *E. coli* was significantly reduced (*P* < 0.05).

#### Fumonisins

Using capillary electrophoresis single-stranded conformation polymorphism (CE-SSCP), it is shown that fumonisins decreased the fecal microbiota SSCP-profiles similarity of the fumonisins-treated swines, compared to the untreated control group. The results indicated that there is an increase in the diversity of microbiota. The balance of the digestive microbiota was transiently but markedly affected after 63 days of chronic exposure to fumonisin, which consists of a mixture of FB1 and FB2 (Burel et al., 2013).

#### Ochratoxin A

A study investigating the effect of OTA on gut microbiota using bioreactors has been carried out by Ouethrani et al. (2013), in which each bioreactor represents different parts of the adult human gut (Ouethrani et al., 2013). Based on the study, the gut microbiota degradation of OTA and microbiota diversity alteration were observed only at the descending colon after 1-week exposure to OTA. PCR-TTGE targeting *Lactobacilli* populations showed that *Lactobacillus reuteri* present during the start-up period, was permanently disappeared at the end of the OTA treatment period accompanied by some minor changes in the bifidobacteria population. The alteration was explained by a significant reduction in acetic, butyric and total SCFA concentration (*P* < 0.05). The reduction of beneficial microbes, lactobacillus and bifidobacteria indicated that the OTA shifted the microbiota balance and possibly led to impaired immunity.

*In vivo* study in rat demonstrated that OTA treatment decreased the diversity of the gut microbiota (Guo et al., 2014). The authors also reported that the relative abundance of Lactobacillaceae was increased whereas the Bacteroidaceae was decreased. Moreover, at the genus level, the OTA decreased the population of Bacteroides, Dorea, Escherichia, Oribacterium, Ruminococcus, and Syntrophococcus, while increased the number of Lactobacillus. The results showed Lactobacillus was more resistant to OTA and Lactobacillus may play a role in OTA detoxification process. Apart from that, it was also reported that the total facultative anaerobes were increased by the OTA treatment. In fact, the increase in facultative anaerobes was often observed in individuals with health complication (Shimizu et al., 2011) as well as in the elderly (Rudi and Avershina, 2015). This may further suggest that the OTA may cause negative effects on the host health via gut microbiota modulation.

#### Aflatoxin

In contrast to the intense research on AFB1 untoward effects, little information is available in regard to the outcomes of AFB1 on the gut microbiota. The findings from Wang et al. (2015) suggested that AFB1 could alter the gut microbiota in a dose-dependent manner. AFB1 decreased phylogenetical diversity but increased evenness of community composition. Although there was no changes at the phylum level, some lactic acid bacteria were significantly (*P* < 0.05) reduced in the presence of AFB1 (Wang et al., 2015). The reduction of LAB in animals treated with a lower dosage of AFB1 explains the severe immune malfunction induced by lower dosage of AFB1 (Qian et al., 2014).

A recent study showed that AFB1 at a dosage level of 1 ppm significantly (*P* < 0.05) reduced total LAB in the broiler. In contrast, the total number of Gram-negative bacteria and LAB were significantly (*P* < 0.05) increased in the group of broiler fed with 1.5 and 2 ppm of AFB1 (Galarza-Seeber et al., 2016). Besides, it has also been reported that 2.5 ppm of AFB1 increased the production of total volatile fatty acids in broilers, which suggested the association of higher dosage of AFB1 with a higher prevalence of LAB in the intestine (Kubena et al., 2001). Interestingly, a separate study showed greater numbers of bacterial mutants were recovered from mice exposed to AFB1 (Rowland, 1988). The data implies that the genotoxic effects of AFB1 not only affecting the host, but the gut microbiota as well.

#### Combination of mycotoxins

On the other hand, the effect of a combination of mycotoxins on the modulation of gut microbiota has also been investigated. The exposure of gilts to ZEA and DON was found to pose an adverse impact on mesophilic aerobic bacteria. In particular, the amounts of *C. perfringens, E. coli*, and other bacteria in the family *Enterobacteriaceae* were reduced significantly after the 6th week of the experiment (*P* < 0.05). Nevertheless, the biodiversity of microorganisms in the gut was increased. Apart from that, an increase in the metabolism of amino acid by the gut microbiota was also observed. It was suggested that the increased metabolism of amino acid may be detrimental due to the formation of biogenic amines and procarcinogenic compounds (Piotrowska et al., 2014). Besides, a study confirmed AF and fumonisin mixture increased Shiga Toxin-producing *E. coli* (STEC) level in fecal (Baines et al., 2013). The composition of STEC-secreted cytotoxin was affected as reflected in the elevated concentration of intracellular Ca^2+^ with a corresponding increase in cytotoxicity. Mycotoxins are capable of altering the microbial balance of the intestine. Furthermore, the possible pathway proposed is via oxidative stress induced by mycotoxins (Vinderola and Ritieni, 2014). Nonetheless, the mechanisms by which mycotoxins affect the gut bacterial composition however remain unclear.

## The role of gut microbiota in the development of mycotoxicosis: HCC

**Figure 1:**
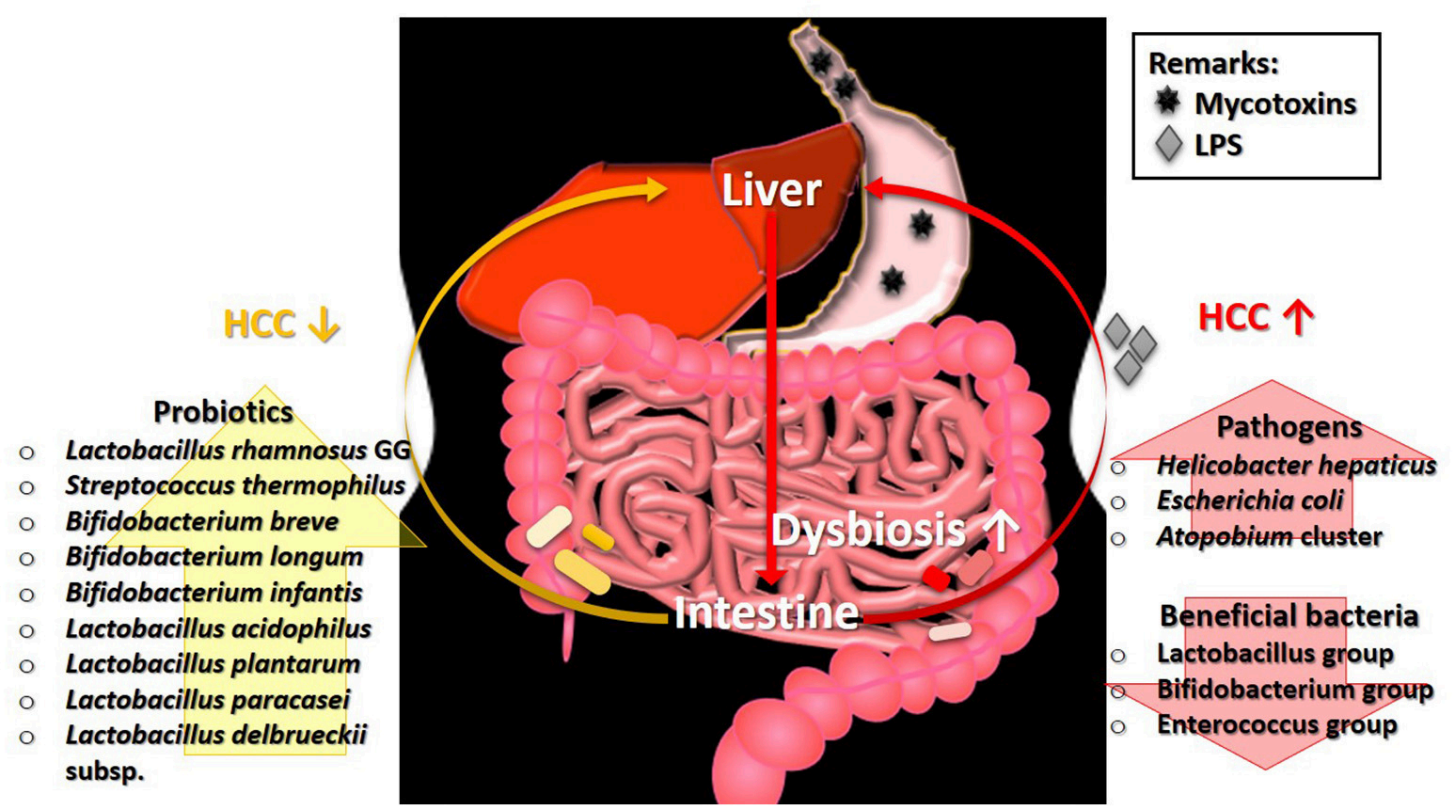
The involvement of gut microbiota in the pathogenesis of HCC. Ingestion of mycotoxin-contaminated foods induces HCC, which eventually leads to the intestinal dysbiosis. The perturbation of microbial balance in the intestine causes a decrease of beneficial gut bacteria. Without the protection from beneficial bacteria, the growth of pathogens will expand rapidly and produce high level of LPS. The presence of LPS exacerbates the condition of HCC. Restoration of gut microbiota balance via intake of probiotics can alleviate the tumorigenic effects in HCC. HCC, hepatocellular carcinoma; LPS, lipopolysaccharide.

Chronic mycotoxicosis, such as HCC results from a high dosage of mycotoxins' contamination. Such pathogenesis generally involves the formation of DNA adducts, regulation of DNA methylation, and alteration of gene expression (Dai et al., 2017; Zhu et al., 2017). Interestingly, gut microbiota perturbation is found to be one of the factors influencing mycotoxin-induced HCC and its association is described in Figure 1. The development of HCC in mice induced by a combination of diethylnitrosamine (DEN) and hepatotoxin carbon tetrachloride (CCl4), a model that features several characteristics of chronically injured livers in which human HCC mostly arises, is prevented via gut sterilization. The same study also showed that mice that were grown in specific GF conditions demonstrated fewer and smaller tumors as compared with those grown under specific pathogen free (SPF) conditions (Dapito et al., 2012). In a toxic model of hepatocarcinogenesis, Yu L. X. et al. (2010) found that the depletion of host microflora suppresses tumor formation. Treatment of rats with antibiotic targeting gram-negative organisms (polymyxin B and neomycin) markedly reduced the size and number of HCC nodules after injection of DEN, which induces HCC.

Some specific bacterial species are also found to be correlated with HCC development. Studies showed that the intestinal colonization by *Helicobacter hepaticus* induced HCC, and the DNA of *Helicobacter* ssp. is only present in liver biopsies from HCC patients, not in control samples (Gargano and Hughes, 2014). Findings from both animal and human studies demonstrated that liver cirrhosis and HCC stimulate an intestinal dysbiosis as well as a significant increase population of the *E. coli* and *Atopobium* cluster, coupled with a significant (P < 0.05) reduction in the percentages of beneficial microbes such as Lactobacillus group, Bifidobacterium group, and Enterococcus group (Zhang et al., 2012). Besides, hepatocarcinogenesis is found to be related to the increased lipopolysaccharides (LPS) levels which are commonly produced by pathogens in several studies (Zhang et al., 2012).

Probiotics are known for their roles in gut health and microbiota restoration. In addition, many strains of probiotics possess the ability to reduce the level of mycotoxins, particularly via binding. Treatment with probiotics mixture, Prohep [*Lactobacillus rhamnosus* GG, heat-inactivated VSL#3, and viable *E. coli* Nissle 1917 (1:1:1)] successfully relieved the microbial imbalance and hepatic inflammation, which further decreased liver tumor growth (Li et al., 2016). A human study by El-Nezami et al. (2006) demonstrated a statistically significant decrease (up to 55% at 5th week; *P* < 0.05) of urinary AFB-N7-guanine level in the probiotic (*L. rhamnosus* LC705 and *Propionibacterium freudenreichii* subsp. *shermanii*) mixture group compared to the placebo group. Similar finding was found by Mohd Redzwan et al. (2016) where serum AFB1-lys level were significantly lower (*P* < 0·05) in the *Lactobacillus casei* Shirota supplemented individuals. Besides, hepatic transcriptome in AFB1-induced HCC was positively altered by probiotics (Monson et al., 2015). Probiotic supplement reduces the biologically available effective toxic dose of mycotoxin coupled with its gut microbiota normalization ability, offer an effective dietary approach to decrease the risk of liver cancer. As shown in these studies, the restoration of gut microbiota equilibrium offers protection and treatment effects in HCC whereas the occurrence of HCC is linked to the higher abundance of pathogens as illustrated in Figure 1. The linkage of microbiota and HCC is undeniably important to understand the mechanism involved in the pathogenesis of HCC.

## Conclusions

This concise review has attempted to draw together the keyworks to highlight the crucial interaction between mycotoxins, the gut, and the gut microbiota in human and animal health. The mycotoxins and gut microbiota studies have revealed meaningful interactions. The uptake of mycotoxin and subsequent tissue distribution are governed by GI tract absorption, and the presence of microbiota at the GI tract can affect the intestinal barrier causing different (maximal or limited) bioavailability of these fungal compounds. The gut microbiota can vary within the same species, thus different reactions toward mycotoxin can be observed as discussed in this review article. In addition, mycotoxins disrupt the gut microbiota balance, and thereby dysregulate intestinal functions and impair local immune response, which may eventually result in systemic toxicity that leads to chronic mycotoxicosis, HCC. The severity of HCC condition can be positively governed by restoration of gut microbiota balance and gut health via probiotics administration. Probiotic which generally helps restore the natural harmony of gut microbiota coupled with its mycotoxins reducing ability could increase its health-promoting value. Regardless, more studies are needed to elucidate the interaction between the gut microbiota and mycotoxin and the implication of such interaction for mycotoxicosis prevention/treatment.

## Author contributions

SM-R and W-P-PL provided the conception and the structure of the article. W-P-PL wrote the draft. SM-R and W-P-PL revised the article and approved the final version to be published.

### Conflict of interest statement

The authors declare that the research was conducted in the absence of any commercial or financial relationships that could be construed as a potential conflict of interest.
